# Validity and reliability of the Persian version of the Patient readiness to engage in health information technology (PRE-HIT) instrument

**DOI:** 10.1186/s12875-022-01665-3

**Published:** 2022-03-19

**Authors:** Reza Safdari, Ping Yu, Sahar Khenarinezhad, Ehsan Ghazanfari Savadkoohi, Zohreh Javanmard, Ala Yousefi, Saeed Barzegari

**Affiliations:** 1 Department of Health Information Management, School of Allied Medical Sciences, Tehran University of Medical Sciences (TUMS), Tehran, Iran; 2 Centre for Digital Transformation, School of Computing and Information Technology, University of Wollongong, Wollongong, Australia; 3 Department of Health Information Management, School of Health Management and Information Sciences, Iran University of Medical Sciences, Tehran, Iran; 4 Instructor, Department of Health Information Technology, Ferdows School of Paramedical and Health, Birjand University of Medical Sciences, Birjand, Iran; 5 Department of Paramedicine, Faculty of Paramedical Sciences, Mazandaran University of Medical Sciences, Sari, Iran

**Keywords:** PRE-HIT, Patient readiness, Health information technology, Psychometric properties, Consumer health information, Public health

## Abstract

**Background:**

The Patient readiness to engage in health information technology (PRE-HIT) is a conceptually and psychometrically validated questionnaire survey tool to measure willingness of patients with chronic conditions to use health information technology (HIT) resources.

**Objectives:**

This study aimed to translate and validate a health information technology readiness instrument, the PRE-HIT instrument, into the Persian language.

**Methods:**

A rigorous process was followed to translate the PRE-HIT instrument into the Persian language. The face and content validity was validated by impact score, content validity index (CVI) and content validity ratio (CVR). The instrument was used to measure readiness of 289 patients with chronic diseases to engage with digital health with a four point Likert scale. Exploratory factor analysis (EFA) and confirmatory factor analysis (CFA) was used to check the validity of structure. The convergent and discriminant validity, and internal reliability was expressed by average variance extracted (AVE), construct reliability (CR), maximum shared squared variance (MSV), average shared square variance (ASV), and Cronbach's alpha coefficient. Independent samples, t-test and one-way ANOVA were used respectively to compare the impact of sex, education and computer literacy on the performance of all PRE-HIT factors.

**Results:**

Eight factors were extracted: health information needs, computer anxiety, computer/internet experience and expertise, preferred mode of interaction, no news is good news, relationship with doctor, cell phone expertise, and internet privacy concerns. They explained 69% of the total variance and the KMO value was 0.79; Bartlett's test of sphericity was also statistically significant (sig < 0.001). The communality of items was higher than 0.5. An acceptable model fit of the instrument was achieved (CFI = 0.943, TLI = 0.931, IFI = 0.944, GFI = 0.893, RMSEA ≤ 0.06, χ2/df = 1.625, df = 292, *P*-value ≤ 0.001). The Cronbach's alpha coefficient achieved a satisfactory level of 0.729. The AVE for all factors was higher than 0.50 except for PMI (0.427) and CIEE (0.463) and also the CR for all factors was higher than 0.7, therefore, the convergent validity of the instrument is adequate. The MSV and ASV values for each factor were lower than AVE values; therefore, the divergent validity was acceptable.

**Conclusion:**

The Persian version of the PRE-HIT was empirically proved for its validity to assess the level of readiness of patients to engage with digital health.

## Introduction

Plagued by the COVID-19 pandemic, the global adoption of digital health has been expedited. For example, telehealth has been widely adopted to bring essential health care to patients while minimising the risk of direct human-to-human exposure [1]. In general, there is an increasing recognition of the contribution of digital health for improving quality of care, reducing medical errors, [2, 3] managing chronic diseases, and improving health service efficiency and reducing costs. Communication over the internet, mobile or computer, between physicians and patients, has many potential benefits [4]. Also, digital health can inform and empower patients to actively engage with planning and managing life style and self-care [5]. However, all these benefits cannot be achieved without consumer readiness [6]; therefore, it is important to understand consumer readiness to engage with digital health [7].

Technology readiness has been conceptualised as the level of willingness, understanding, and skill in using the technology [2, 8]. Assessment of readiness can help designers to design effective digital solutions, i.e., web-based and mobile applications [9]. A range of digital health readiness measurement instruments have been developed (see Table 1). Kayser et al. developed and validated the psychometric property of READHY tool via a questionnaire survey with 305 cancer patients (see Table 1). The instrument assesses patients’ knowledge and skills, readiness and ability to engage with and benefit from healthcare technologies [7]. Hirani et al. (2017) conceptualized and validated the psychometric property of SUTAQ, the questionnaire survey instrument to predict user’s acceptance of telemedicine tools based on their prior experience. SUTAQ also predicts users’ beliefs and behavior with telemedicine tools. Its weakness is a lack of consideration of users’ health knowledge and digital skill [7, 10]. The PERQ includes eight questions that ask about patient’s internet usage, social support, personal abilities, economic status and self-efficacy in using eHealth applications [11]. However, its conceptual and psychometric validity has not been tested. Norman and Skinner developed the eHEALS instrument to measure consumer eHealth literacy and ability to search, use, and evaluate health resources on the internet. Limitations of this instrument include an inability to directly measure consumers’ eHealth skills, and its validity was only tested in 13–21 year olds with high level use of technology, not in adults [12]. Its measurement items need to be revised and further validated [13].

**Table 1:** Comparison of the published instruments for measuring consumer readiness to use digital health

Tool, Author and Year of publication	Goal	Factors	Items	Variance Explained	Limitations
**PERQ**, Jones et al., (2013)	Assess patient readiness to use eHealth tools	**Four factors:** Patients’ perception of (1) provision, (2) their personal ability and confidence, (3) their interpersonal support, and (4) relative costs in using the Internet for health	Nine	Not mentioned	Is not a conceptually and psychometrically validated tool
**eHEALAS**, Norman and Skinner, (2006)	Assess user skill and knowledge with digital solutions	**Single-factor instrument**	10	56%	Can’t measure consumers’ skills directly, presents a limitation in testing with a population that has high rates of information technology familiarity and older adults
**SUTAQ**, Hirani et al., (2016)	Assess the acceptance of telemedicine tools	**Five factors:** perceived benefit, privacy and discomfort, care personnel concerns, kit as a substitution, and satisfaction	22	60.7	Did not consider user’s knowledge and skills about information technologies
**READHY**, Kayser et al., (2019)	Measure consumer readiness to use health technologies	**Five factors:** users’ knowledge and skills (3 items: using technology to process health information; understanding of health concepts and language ability to actively engage with digital services); self-management of disease (2 items: self-monitoring and insight; skills and technique acquisition); perceptions and mindset (4 items: feel safe and in control; motivated to engage with digital services; constructive attitudes and approaches; emotional distress); experience with health technology systems (2 items: access to digital services that work; digital services that suit individual needs); understanding of the extent to which users feel supported by relatives, peers, and health professionals (2 items:, feeling understood and supported by health care providers; social support for health)	65	Not mentioned	Insufficient sample size
**PRE-HIT**, Koopman et al., (2014)	Measure patient readiness to interact with health technologies	**Eight factors:** health information needs, computer anxiety, computer/ internet experience, and expertise, preferred mode of interaction, no news is good news, relationship with doctor, cell phone expertise, and internet privacy concerns	28	Not mentioned	The lack of certain scores to predict use and non-use of technology

*PERQ* patient eHealth readiness questionnaire, *eHEALS* eHealth literacy scale, *SUTAQ* service user technology acceptability questionnaire, *READHY* readiness and enablement index for health technology, *PRE-HIT* patient readiness to engage in health internet technology

PRE-HIT is a conceptually and psychometrically validated questionnaire survey tool that is built upon eHEALS to measure willingness of patients with chronic conditions to use health information technology (HIT) resources. The instrument has 28 items that are grouped into 8 factors: health information needs (HIN), computer anxiety (CA), computer/internet experience and expertise (CIEE), preferred mode of interaction (PMI), no news is good news (NNGN), relationship with doctor (RWD), cell phone expertise (CPE), and internet privacy concerns (IPC) (see Table 2). It uses a 4-point Likert scale to measure each item. The test score for the PRE-HIT test ranges from the lowest of 28 to the highest of 112. The weakness of the instrument is a lack of a clear indicator to predict use or non-use of HIT [9].

**Table 2:** Definition of the PRE-HIT factors

Factor	Items	Definition
Health Information Need (HIN)	5	A person’s recognition that own knowledge is inadequate to satisfy the person’s health goal within a certain context/situation at a specific point in time (Ormandy, 2011)
Computer Anxiety (CA)	4	A person’s fear of inability to use computer technology or that it may cause damage as the result of computer usage (Marcoulides, 1989)
Computer/Internet Experience, Expertise (CIEE)	4	Ability of a person to resolve computer/internet usage problems that they might run into (Koopman et al., 2014)
Preferred Mode of Interaction (PMI)	5	Preferred model of contact with doctor, looking up health concerns, and trusted source of health information (Koopman et al., 2014)
No News is Good News (NNGN)	3	Seeking information on the internet could lead a person to encounter more information than they need, and some of that information could be distressing (Koopman et al., 2014)
Relationship with Doctor (RD)	3	It refers to a person's trust of physicians as a source of health information and to handle the person’s health (Koopman et al., 2014)
Cell Phone Expertise (CPE)	2	Going online or text people using a cell phone (Koopman et al., 2014)
Internet Privacy Concern (IPC)	2	Concern about own information transmitted over the internet, which may be acquired by unauthorized third party (Koopman et al., 2014)

Not limited to examining patients’ eHealth literacy, i.e., computer and internet literacy, media literacy, and desire to search for information, the PRE-HIT also covers broader factors that may influence patients’ decision to adopt digital health, including information needs, privacy considerations, IT usage experience, information source, and preferred interaction and motivation method. [9]. In comparison with other similar instruments, we believe that PRE-HIT is the most comprehensive and useful instrument for examining patients’ readiness to engage with digital health.

### Objective

This study aims to translate, implement, and validate the Persian version of the PRE-HIT instrument.

## Methods

This research was conducted in three steps. First, the original PRE-HIT instrument was translated into a Persian version. Then a cross-sectional questionnaire survey was conducted to collect empirical data from the patients using the Persian version of the PRE-HIT instrument. In Step 3, exploratory and confirmatory factor analysis was conducted to test the structural validity of the instrument.

### Step 1. Translation of the PRE-HIT instrument into the Persian version

The translation task was completed in three sub-steps: forward translation, face and content validation, and back translation.

### Forward translation

At first, items were translated by one translator, a specialist in digital health. Translation considered cross-cultural and conceptual equivalence rather than linguistic equivalence for words and phrases to ensure the translated version is concise, simple and fits with Persian language and culture.

### Face and content validity

The expert panel consisted of four faculty members; two from the nursing faculty, one expert in health information management and one from medical informatics. All were familiar with psychometric studies. The panel evaluated the face validity and content validity of the Persian version of the PRE-HIT instrument both qualitatively and quantitatively. Qualitative face validity was assessed by identification of problems and ambiguity in translation, and time required to answer a question. The suggestions of every expert were taken to change words to improve clarity or modify sentences to correct grammatical errors, to simplify expression without losing meaning, or using more appropriate words. Quantitative face validity was assessed using the Impact Score, which was calculated by the formula of *frequency* × *importance for each item*. The experts ranked each item on a 5 point Likert Scale ranging from very important (Score 5) to least important (Score 1). Frequency referred to the percentage of experts who gave an item a score of 4 or 5. Importance referred to the mean score of each item [14]. An item would be kept if its Impact Score was larger than or equal to 1.5. Quantitative content validity was evaluated by the content validity index (CVI) and content validity ratio (CVR). We used the CVI to examine the relevance of each item with the PRE-HIT construct. The expert panel used a 4-point Likert Sale to rate an item (1 = not relevant, 2 = somewhat relevant, 3 = quite relevant, 4 = highly relevant). CVI score was calculated by the following formula. Items with the CVI score greater than or equal to 0.79 were retained [15].

$$CVI=number\;of\;experts\;giving\;a\;rating\;of\;\mathit"highly\mathit\;relevant\mathit"\mathit\;for\;an\;item/total\;number\;of\;experts$$

The necessity of the items in the PRE-HIT construct was calculated by the Lawshe test [16]. For this, the expert panel scored an item by 3-point Likert Scale, ranging from essential, useful but not essential, and not necessary. The CVR score was calculated by the following formula. Items with the CVR greater than or equal to 0.49 were retained [15].

$$CVR=(Ne-N/2)/(N/2)$$

where Ne is the number of experts identifying an item as “essential” and N is the total number of experts.

No cross-cultural and conceptual problems were found. All items achieved the impact scores and all items were equal to or greater than 1.5, the CVI and CVR scores above 0.79 and 0.49, respectively; therefore, their face and content validity were proved.

### Back-translation

The Persian version of the questionnaire was translated back to English by an independent translator, who did not know the questionnaire. The translator was an expert in Health Informatics. Attention was paid to conceptual and cross-cultural equivalence. Afterwards, the translator and the research team discussed the English translation and reached agreement on its validity.

### Step 2. Cross-sectional questionnaire survey

#### The design of the questionnaire

The questionnaire was comprised of two parts. The first part asked questions about demographic characteristics such as age, sex, level of education, and ownership of the International Computer Driving Licence (ICDL). The second part contained the 4-point Likert Scale questions asking about the PREHIT items.

### Sample size calculation

Because factor analysis (FA) would be applied to investigate the psychometric properties of the PRE-HIT instrument, for valid FA, 5 to 10 samples are required to address a question item [17]. As the PRE-HIT has 28 items, 280 questionnaire responses were required. Three hundred patients with chronic diseases were selected and invited to participate by doctors based on inclusion and exclusion criteria, and 289 samples participated and responded to the questionnaire.

### Study population

#### Inclusion and exclusion criteria

Patients who met the following inclusion criteria were recruited: 1) aged 18 years or over; 2) being conscious and not having serious complications such as mental disorders; and 3) able to read in Persian.

#### Participant recruitment

The doctors in the teaching hospitals in Cardiology, Dermatology, Gastroenterology, and Internal Medicine recruited patients with chronic diseases at discharge and inpatients with stable conditions. The questionnaires were handed to the patients directly by the researchers. The aim of the study, its voluntary nature, and assurance about anonymity of results in any resulting publications were orally explained by the researchers. Informed consent was obtained before distributing the questionnaire. Data collection was conducted during March 1 to August 1, 2020.

### Step 3. Data analysis

#### Exploratory and confirmatory factor analysis

To evaluate the construct validity, exploratory factor analysis (EFA) was conducted in SPSS version 19. Due to the significant correlation between items, the Promax rotation was used to extract the latent factors. Eigenvalue ≥ 1 was used to identify the factors. Explained variance of each factor and cumulative explained variance for the entire survey were obtained. The Kaiser–Meyer–Olkin (KMO) index was checked for proportion of variance in the variables that might be caused by the underlying factors. Bartlett’s Test of Sphericity was conducted to check redundancy between the variables. If an item had a Communality value below 0.5, it would be deleted [15].

Confirmatory factor analysis (CFA) with maximum likelihood was applied to evaluate the goodness of fit of the extracted structure by EFA. The goodness of fit indices such as Comparative Fit Index (CFI ≥ 0.90), Tucker–Lewis Index (TLI ≥ 0.90), Root Mean Square Error of Approximation (RMSEA ≤ 0.06), Chi-square/Degree of Freedom (CMIN/DF ≤ 3), and Goodness of Fit Index (GFI ≥ 0.90) were checked [18]. Factor loading for each item was also examined. Analysis was performed in Amos version 19.

### Convergent and discriminant validity and internal reliability

Convergent and discriminant validity are two aspects of construct validity. Convergent validity, evaluated through average variance extracted (AVE), and construct reliability (CR), ensure the relationship between two theoretically related factors of a construct. The CR and AVE for the factors of a construct should exceed 0.70 and 0.50 respectively. Discriminant validity, evaluated through maximum shared squared variance (MSV), and average shared square variance (ASV), ensures there is no relationship between two theoretically unrelated factors. For discriminant validity, the AVE value must be higher than the two MSV and ASV values [19]. Also, internal reliability was assessed by Cronbach's alpha coefficient with value higher than 0.7 indicating an acceptable level of reliability [20].

### Comparison of the mean value of the factors between different groups

The criteria of sex, computer literacy, and education level were used for demographic groupings. The Shapiro–Wilk test was conducted to assess the normality of the distribution of data. One-way ANOVA test was conducted to compare means of each factor in different education levels. Independent samples t-test and Mann–Whitney U test were conducted to compare different demographic groups’ performance on each factor.

## Results

Three hundred patients with chronic diseases were invited, 289 (96.33%) participated with informed consent, and returned the questionnaire responses. The mean age of the respondents was 44.76 ± 5.85 years (range: 36–64 years). The majority were females (53.8%, *n*  =  155), and most had no ICDL certificate (84.78%, *n*  =  245). Women had significantly higher scores than men in HIN (Women: 3.10 ± 0.66; Men: 2.78 ± 0.52, *p* <  = 0.001) and NNGN (Women: 2.80 ± 0.63; Men: 2.61 ± 0.64, *p* = 0.019). Conversely, the mean scores of the CPE in men were significantly higher than that in women (Women: 3.54 ± 0.63; Men: 3.56 ± 0.65, *p* = 0.026). There was a positive association between level of education and CIEE, PMI, and CPE. The mean scores of the HIN, CIEE, PMI, and CPE factors were significantly higher in people who had ICDL certificates than otherwise (see Table 3).

**Table 3:** Comparison of the mean scores of each factor between groups

*P*-value	ICDL Certificate		*p*-value	Education level			*p*-value	Gender		Mean (S.D.)	Factors
	No (245)	Yes (44)		H^§^	B ^‡^	D^†^		F (201)	M (88)		
0.035^a^	2.96	3.12	D-B (0.989) D-H (1) B-H (0.990)	3.01	3	3.01	< = .001^a^	3.10	2.78	3.00 (0.58)	HIN
0.057	2.49	2.33	D-B (0.999) D-H (0.444) B-H (0.379)	2.38	2.49	2.50	0.331	2.47	2.39	2.45 (0.63)	CA
< = 0.001^a^	3.13	3.48	D-B (0.09) D-H (< = .001^a^) B-H (< = .001^a^)	3.48	3.11	2.93	0.357	3.20	3.27	3.22 (0.57)	CIEE
0.014^a^	2.67	2.82	D-B (0.174) D-H (0.019^a^) B-H (0.593)	2.78	2.72	2.59	0.811	2.72	2.70	2.71 (0.46)	PMI
0.353	2.72	2.80	D-B (0.745) D-H (0.855) B-H (0.966)	2.74	2.77	2.69	0.019^a^	2.80	2.61	2.74 (0.64)	NNGN
0.110	3.21	3.31	D-B (0.994) D-H (0.740) B-H (0.602)	3.20	3.27	3.26	0.756	3.24	3.22	3.24 (0.51)	RWD
0.021^a^	3.43	3.61	D-B (0.267) D-H(0.003^a^) B-H (0.074)	3.62	3.45	3.28	0.026^a^	3.54	3.56	3.48 (0.63)	CPE
0.746	2.65	2.68	D-B (0.558) D-H(0.439) B-H (0.982)	2.62	2.63	2.75	0.230	2.62	2.73	2.65 (0.74)	IPC

^a^Statistically significant difference in mean; *†* Diploma, * ‡* Bachelor, *§* Master of science and higher

The KMO value was 0.79, suggesting that a certain proportion of variance in digital health readiness is caused by underlying factors. Bartlett's test of sphericity was statistically significant (sig < 0.001), suggesting minimal redundancy between the factors, thus the data set was suitable for EFA. The communality of items was higher than 0.5 (ranged from 0.550 to 0.877), suggesting that each item loaded significantly only on one factor. The factor loading of each item was ≥ 0.6, except for item 8 (0.584) (see Table 4). This suggests high relevance of the items in explaining the corresponding factor. Eight factors were extracted by EFA, which explained 69% of the total variance. In descending order of the variance explained, the factors were HIN = 20.34, CA = 12.68, CIEE = 9.35, PMI = 7.08, NNGN = 6.08, RWD = 5.08, CPE = 4.72, and IPC = 4.07%, respectively.

**Table 4:** Factor loadings of each item and variance explained by each factor

Factor	Items	Factor loading	% of Variance
1 Health Information Need (HIN)	Q24: I would use the internet to look up things so that I wouldn’t worry about them anymore	0.769	20.34
	Q25: I would use the internet to look up information about herbs and/or supplements	0.826	
	Q26: I would use the internet to look up symptoms	0.857	
	Q27: I would use the internet to search for information about my health	0.831	
	Q28: I would use the internet to find information about medications	0.842	
2 Computer Anxiety (CA)	Q15: Using the computer is boring for me	0.827	12.68
	Q21: When I use the Internet, I would get frustrated and tired with the amount of information I found about health	0.706	
	Q22: I think that searching for information would be stressful	0.749	
	Q23: If I went on the internet, I would find sorting through information to be too time consuming	0.769	
3 Computer/Internet Experience, Expertise (CIEE)	Q14: When I use a computer, I would be able to figure out most problems that I might run into	0.642	9.35
	Q16: When I use a computer, I would have access to the internet	0.752	
	Q17: Using the internet is easy for me	0.716	
	Q18: Using the email service is easy for me	0.799	
4 Preferred Mode of Interaction (PMI)	Q5: I trust the internet as a source for health information	0.675	7.08
	Q8: Looking up health concerns on the internet is more convenient for me than contacting a doctor’s office	0.585	
	Q9: I prefer calling my doctor’s office to emailing them	0.664	
	Q10: I would email my doctor because it is easier than going to the office	0.732	
	Q11: Looking up information online about medications is easier than asking my doctor	0.620	
5 No News is Good News (NNGN)	Q2: People today want to know more about their health	0.859	6.08
	Q3: Regarding my health, I agree with the statement “No news is good news.”	0.802	
	Q6: I am concerned about what I might find on the Internet about my health problems	0.841	
6 Relationship with Doctor (RWD)	Q1: I let my doctor to control the details of my health	0.738	5.08
	Q4: Doctors are my most trusted source of health information	0.875	
	Q7: When I have a health concern, my first step is to contact my doctor’s office	0.787	
7 Cell Phone Expertise (CPE)	Q12: I go online using my cell phone	0.826	4.72
	Q13: I use my cell phone to text people almost every day	0.717	
8 Internet Privacy Concerns (IPC)	Q19: When I use the internet, I would be very concerned about giving any personal information	0.909	4.07
	Q20: When I use the internet, I would be concerned it would lead to invasions of my privacy	0.899	

**Fig. 1:**
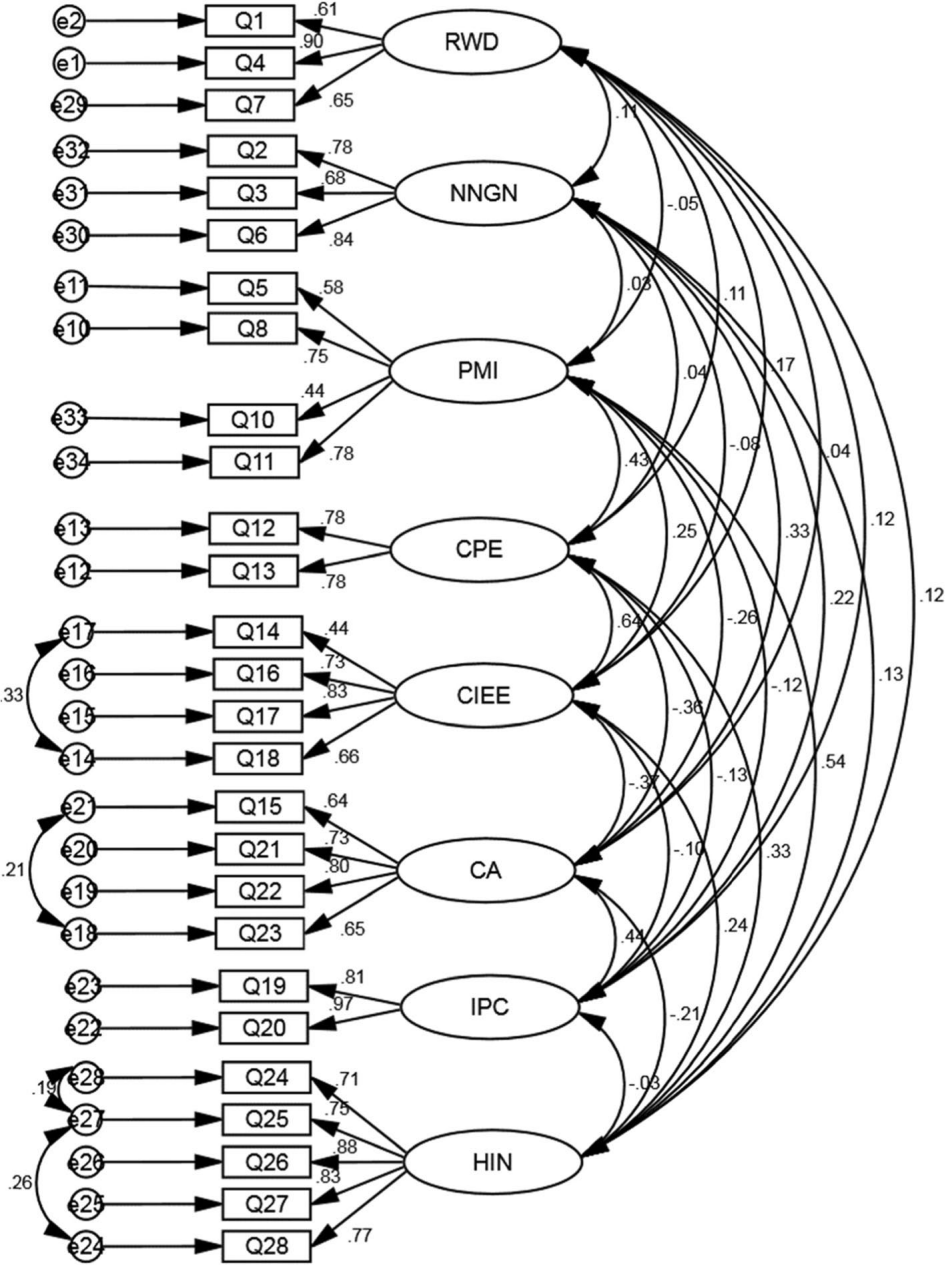
The final fitted model by confirmatory factor analysis

Confirmatory factor analysis confirmed the goodness-of-fit of the factor structure with all of the goodness of fit indices on the favorable threshold (see Fig. 1). The CFI, TLI, IFI, GFI, and RMSEA indices were at the acceptable threshold (CFI = 0.943, TLI = 0.931, IFI = 0.944, GFI = 0.893, RMSEA ≤ 0.06, χ2/df = 1.625, df = 292, *P*-value ≤ 0.001)

One item (Item 9) was removed from the tool due to low factor loading (0.39). After deleting this item, the internal consistency as assessed by the Cronbach's alpha coefficient achieved a satisfactory level of 0.729 (see Table 5). The Cronbach's alpha coefficient for each factor, including RWD = 0.750, NNGN = 0.807, PMI = 0.733, CPE = 0.747, CIEE = 0.770, CA = 0.813, IPC = 0.880, and HIN = 0.897, was above 0.70; therefore, the internal consistency of the questionnaire was optimal. The results of convergent and discriminant validity, internal consistency, and CR are presented in Table 5. The AVE for all factors is higher than 0.50 except for PMI (0.427) and CIEE (0.463). The CR for factors were higher than 0.7 and ranged from 0.740 to 0.892, which was acceptable. Claes & Larcker (1981) stated that if the AVE of a factor is less than 0.5 but its composite reliability is higher than 0.6, the convergent validity of the construct is adequate [21]. Therefore, the AVE and CR values approve the convergent validity of the PRE-HIT instrument. Also, the MSV and ASV values for each factor were lower than AVE values; therefore, the divergent validity of all factors was acceptable.

**Table 5:** Internal consistency, convergent and discriminant validity, and CR values

Factor	Items	Cronbach’s Alpha if Item Deleted	Item-Total Correlation	Cronbach’s Alpha	CR	AVE	MSV	ASV
1 HIN	Q24	0.888	0.685	0.897	0.892	0.625	0.292	0.076
	Q25	0.873	0.755					
	Q26	0.863	0.803					
	Q27	0.873	0.754					
	Q28	0.875	0.748					
2 CA	Q15	0.768	0.633	0.813	0.802	0.506	0.191	0.098
	Q21	0.776	0.610					
	Q22	0.748	0.670					
	Q23	0.770	0.623					
3 CIEE	Q14	0.765	0.489	0.770	0.767	0.463	0.404	0.101
	Q16	0.708	0.591					
	Q17	0.710	0.608					
	Q18	0.675	0.647					
4 PMI	Q5	0.676	0.527	0.733	0.740	0.427	0.292	0.089
	Q8	0.672	0.533					
	Q9	0.728	0.377					
	Q10	0.693	0.484					
	Q11	0.661	0.559					
5 NNGN	Q2	0.720	0.682	0.807	0.814	0.595	0.109	0.028
	Q3	0.781	0.611					
	Q6	0.703	0.696					
6 RWD	Q1	0.737	0.516	0.750	0.767	0.532	0.028	0.012
	Q4	0.532	0.685					
	Q7	0.697	0.554					
7 CPE	Q12	-	0.608	0.747	0.756	0.608	0.404	0.123
	Q13	-	0.608					
8 IPC	Q19	-	0.786	0.880	0.887	0.799	0.191	0.042
	Q20	-	0.786					

*CR* construct reliability, *AVE* average variance extracted, *MSV* maximum shared squared variance, *ASV* average shared square variance

## Discussion

This study developed and validated the Persian version of PRE-HIT in measuring digital health readiness of Iranian patients with chronic illness. The instrument achieved a satisfactory level of reliability and validity, and factor loading. To the best of our knowledge, this is the first study to examine the psychometric properties of the PRE-HIT instrument, and in only one study, Samadbeik et al. examined the instrument's internal reliability by Cronbach's alpha coefficient. They found that the instrument has optimal internal reliability. Based on our results, the Cronbach's alpha coefficient for the instrument and each factor was above 0.70 and achieved the satisfactory level; therefore, the internal reliability of the questionnaire was optimal. Our finding was in accordance with the Samadbeik et al. study. In addition, the number and structure of the extracted factors were in accordance with Koopman's study [9]. Thus the Persian version of the PRE-HIT is valid to measure readiness of Iranian patients in engaging with digital health.

There are mixed findings in comparing computer literacy levels between men and women. Women had a significantly higher level of health information needs than men, as found by Stewart et al. (2004) in 635 Canadian adults. Their results showed that women were keener to seek information on angina (1.77 times) and blood pressure (1.57 times) [22]. Previous studies also found that women were more likely than men to use the internet to access health information [23, 24]. Also, women with chronic medical conditions were more likely to search health information [25].

Joiner found that computer use efficiency in women is lower than in men [26]. This may explain the higher level of computer anxiety we observed in women than in men, although not statistically significant. Dyck et al. reached a similar finding [27]. Conversely computer/internet experience and IT expertise were higher in men than in women, which is consistent with the previous findings [26, 28]. However, this gender difference was not supported by Samadbeik et al. [29]. No difference was found in “relationship with doctor” across educational levels and gender, which is in agreement with the finding of Cooper-Patrick [30].

Both male and female patients held moderate levels of privacy concerns, which is different from the finding of Youn that females had a higher level of privacy concerns [31]. Similar to Atherton et al. (2012) and Hanauer et al. (2009) [4, 32], this study found that the level of digital readiness was equally high in men (mean = 2.86) and women (mean = 2.94).

## Conclusion

The Persian version of the PRE-HIT is a reliable and valid tool to evaluate and compare the level of digital readiness of patients with chronic illness. This tool is useful for policy makers and healthcare organisations to measure patients’ digital readiness, and to inform options and strategies to introduce consumer digital health solutions for patient self-management of disease.

## Data Availability

Data available on request from the authors.
